# Neuropilin-1 Acts as a Receptor for Complement Split Products

**DOI:** 10.3389/fimmu.2019.02209

**Published:** 2019-09-13

**Authors:** Claire Battin, Annika De Sousa Linhares, Wolfgang Paster, David E. Isenman, Markus Wahrmann, Judith Leitner, Gerhard J. Zlabinger, Peter Steinberger, Johannes Hofer

**Affiliations:** ^1^Division of Immune Receptors and T Cell Activation, Center for Pathophysiology, Infectiology, and Immunology, Institute of Immunology, Medical University of Vienna, Vienna, Austria; ^2^Department of Clinical Cell Biology and FACS Core Unit, Children's Cancer Research Institute (CCRI), Vienna, Austria; ^3^Departments of Biochemistry and Immunology, University of Toronto, Toronto, ON, Canada; ^4^Division of Nephrology and Dialysis, Department of Internal Medicine III, Medical University Vienna, Vienna, Austria; ^5^Division of Clinical and Experimental Immunology, Center for Pathophysiology, Infectiology, and Immunology, Institute of Immunology, Medical University of Vienna, Vienna, Austria

**Keywords:** complement split products, neuropilin-1, complement receptors, C4d, C3d, iC3b

## Abstract

Complement split products (CSPs), such as the fragments C4d and C3d, which are generated as a consequence of complement regulatory processes, are established markers for disease activity in autoimmunity or antibody-mediated graft rejection. Since immunoglobulin-like transcript 4 (ILT4) was previously shown to interact with soluble CSPs, but not with CSPs covalently-bound to target surfaces following classical complement activation, the present study aimed to identify novel cellular receptors interacting with covalently-deposited CSPs. By applying an unbiased screening approach using a cDNA mammalian expression library generated from human monocyte-derived dendritic cells and probed with recombinant human C4d, we identified neuropilin-1 (NRP1) as a novel receptor for C4d, C3d, and iC3b. NRP1, a highly conserved type 1 transmembrane protein, plays important roles in the development of the nervous and cardiovascular system as well as in tumorigenesis through interaction with its established binding partners, such as vascular endothelial growth factor (VEGF) and semaphorin 3A (Sema3A). NRP1 is also expressed on immune cells and serves as a marker for murine Tregs. Although NRP1 contains domains homologous to ones found in some complement proteins, it has not been linked to the complement system. We demonstrate that binding of C4d to NRP1 expressing cells was dose-dependent and saturable, and had a K_D_ value of 0.71 μM. Importantly, and in contrast to ILT4, NRP1 interacted with CSPs that were covalently bound to target surfaces in the course of complement activation, therefore representing a classical complement receptor. The binding site of CSPs was mapped to the b1 domain of the coagulation factor V/VIII homology domain of NRP1. Taken together, our results demonstrate a novel role for NRP1 as a receptor for CSPs deposited on surfaces during complement activation. Further work is required to elucidate the functional consequences of the NRP1-CSP interactions in immunity.

## Introduction

The complement system mediates robust first line defense mechanisms against invading pathogens and participates in the elimination of cellular and humoral debris (1). It functions as a dynamic network in which diverse complement split products (CSPs) are involved in immune homeostasis and surveillance, but also in the modulation of T- and B- cell responses (2). A signature feature of the pivotal complement split products C3b and C4b is that they covalently tag their target surfaces via a thioester-mediated transacylation onto target hydroxyl and amino groups (3). Their intrinsic thioester, formed between the side chains of nearly adjacent cysteine and glutamine residues located within the respective thiol ester-containing domain (4) of C3 and C4, is buried at a domain interface within the native molecules, but becomes accessible to target nucleophiles during the dramatic conformational changes following the primary proteolytic cleavage events of these molecules during complement pathway activation (5, 6). This covalent tagging, as well as the subsequent immunomodulatory and effector functions of the complement system, is initiated by three distinct pathways: the classical, the lectin and the alternative pathway. Each of the three pathways depends on different molecular patterns represented by antigens or altered self-structures for their initiation. The classical and lectin pathways are initiated by antigen-antibody and by mannan-binding lectin, or ficolin, interactions on microbial surfaces, respectively. In contrast, the alternative pathway does not depend upon a specific pattern-, or antigen-sensing initiation molecule, but rather it continuously maintains a so-called “tick-over” level of activity that can result in the arbitrary covalent deposition of C3b on any target surface, which in turn allows for the assembly of an alternative pathway C3 convertase on that target. However, as described below, stringent host regulatory mechanisms only permit propagation of the pathway when the convertase is present on foreign, or modified-self (e.g., cellular debris) targets, but not on host targets (7).

Host protection is mediated by a combination of cellular and humoral complement regulatory proteins (8). They act in concert by disrupting C3- and C5-convertase assembly and function through both their convertases subunit decay-dissociation properties and their factor I-cofactor activities, the latter leading to the further enzymatic cleavage of the primary CSPs, C4b, and C3b, and the loss in their ability to function as subunits of the classical and alternative pathway C3 convertases, respectively. Additionally, host membranes are protected from the terminal lytic complement complex by the host membrane molecule CD59/protectin. The broadly expressed cellular complement regulatory molecules that regulate the C3- and C5-convertase molecules of both the classical and alternative pathways are membrane cofactor protein (MCP; CD46) and decay-accelerating factor (DAF; CD55). Additionally, complement receptor 1 (CR1, CD35), which is primarily expressed on circulating blood cells, possesses both factor I-cofactor and decay accelerating activities. The membrane-associated regulators act in concert with their fluid-phase counterparts, namely factor H, C4b-binding protein (C4BP), and factor I. The factor I-mediated cleavages of C4b and C3b result in the generation of a number of CSPs that remain covalently-bound to target surfaces, including C4d, iC3b, C3dg. The latter CSP may be trimmed by other serum proteases to remove the N-terminal “g” segment and yield the still target-attached limit fragment C3d. The fragments C4d and C3d correspond approximately to the boundaries of the structurally-defined TED domain (9). Some of these CSPs promote immune activation and contribute to the clearance of pathogenic molecules and apoptotic cells from circulation by acting as ligands for cellular complement receptors. Most of these complement receptors (CR1/CD35; CR2/CD21; CR3/CD11b-CD18; CR4/CD11c-CD18), and their specific CSP ligands, were identified several decades ago, whereas an additional receptor for C3b and iC3b was discovered more recently, termed complement receptor of the immunoglobulin family (CRIg) (10). CRIg was demonstrated to play important roles in the clearance of C3-opsonized pathogens by Kupffer cells in the liver, but also as a potent inhibitor of the alternative pathway of complement activation (10–12). More recently, using an unbiased screening approach based on eukaryotic expression cloning, we identified the first cellular receptor for C4d, namely immunoglobulin-like transcript-4 (ILT4), also known as leukocyte immunoglobulin-like receptor subfamily B member 2 (LILRB2) (13). ILT4, and to a lesser extend also a variant of the polymorphic ILT5 molecule (ILT5v2) were shown to interact with other CSPs, such as C4b, C3d, C3b, and iC3b (13). However, unlike “classical” complement receptors, ILT4 did not appear to interact with CSPs physiologically deposited to surfaces during complement activation via its intrinsic thioester. Instead, this receptor might represent a scavenger-type endocytic receptor for soluble C4d and possibly other CSPs (13).

In the present study, we screened a cell pool expressing a retroviral cDNA library generated from human monocyte-derived dendritic cells (moDCs) and identified neuropilin-1 (NRP1), also known as CD304 and blood dendritic cell antigen-4 (BDCA4), as a novel cellular complement receptor for C4d, C3d, and iC3b. NRP1, a well-described receptor for vascular endothelial growth factor (VEGF) and semaphorins, has been implicated to be involved in various biological processes including angiogenesis, neuronal development, cell survival, migration, and tumor-invasion (14–16). We provide evidence for a classical receptor-ligand interaction between NRP1 and both isoforms of human C4d, namely C4Ad, and C4Bd. Moreover, we succeeded in mapping the binding site for CSPs on NRP1 demonstrating that, unlike ILT4, NRP1 acts as a “classical” complement receptor for CSPs covalently deposited on target surfaces as a consequence of complement pathway activation.

## Materials and Methods

### Cell Culture, Antibodies, and Reagents

Murine thymoma BW5147 (referred to as BW throughout this work) cell line and Jurkat cell line (JE6.1) were cultured as previously described (13, 17). For detection of ectopically expressed surface NRP1, an unlabeled mouse monoclonal (clone 446921) and a sheep polyclonal antibody (R&D systems, Minneapolis, MN, USA) were used. In addition, NRP1-PE (clone 12C2), mNRP1-PE (clone 3E12), and iC3b-PE (clone 3E7/C3b) antibodies were obtained from Biolegend (San Jose, CA). PE-conjugated ILT4 antibody was purchased from Becton Dickinson (Franklin Lakes, NJ). Unlabeled monoclonal C4d-specific antibody (clone CL314) and C4c-specific antibody (clone 10-12) were purchased from Quidel (San Diego, CA). C3d-specific antibody (clone 7C10) was obtained from Abcam (Cambridge, UK). Recombinant human (rh)-CTLA-4-immunoglobulin (Ig), rh-ILT4-Ig, and rh-NRP1-Ig (c.) (amino acids 1-644) fusion proteins were purchased from R&D systems (Minneapolis, MN) or Creative Biomart (Shirley, NY), respectively. Rh-control-Ig (rh-Ctrl-Ig; CD5 leader fused to human IgG-Fc part), rh-CRIg-Ig, rh-NRP1-CUB-Ig (amino acids 1-264 of Uniprot O14786), and rh-NRP1-Ig (amino acids 1-593 of Uniprot O14786) fusion proteins were produced in house as previously described (18). iTag MHC (major histocompatibility complex) class I human tetramer-streptavidin (SA)-PE HLA-A-24:02 (CMV QYDPYAALF) and HLA-B-08:01 (CMV ELRRKMMYM) were purchased from Beckman Coulter (Fullerton, CA). HLA-A-02:01 (CMV LLFGVPVYV) and HLA-A-02:01 (CMV CLGGLLTMV) tetramer-SA-APC were obtained from the NIH-Tetramer Core Facility, Atlanta, GA. For the detection of biotinylated molecules, streptavidin-PE was used from ebioscience (San Diego, CA). PE-conjugated goat anti-mouse IgG (Fcγ-specific), unlabeled goat-anti-mouse IgG (H+L), APC-conjugated goat anti-human (Fcγ-specific), PE-conjugated donkey anti-goat (H+L), DyLight 649-conjugated donkey anti-mouse (Fcγ-specific), and HRP-conjugated goat anti-human (Fcγ-specific) were purchased from Jackson ImmunoResearch Laboratories (West Groove, PA, USA). Hemolysin (amboreceptor) was obtained from Siemens Healthcare Diagnostics Products (Marburg).

### Flow Cytometry

Flow cytometric data acquisition was performed using FACSCalibur^TM^ or LSRFortessa^TM^ (both BD Biosciences, San Jose, CA). Data was analyzed using FlowJo software (version 10.0.8., Tree Star, Ashland, OR). Sorting of high surface molecule expressing cells was carried out with a Cell Sorter SH800 (Sony Biotechnology Inc., San Jose, CA).

### Complement Split Products

Rh-C4Ad, rh-C4Bd, and rh-C3d were expressed and purified as previously described (19, 20). Ih-C4d was isolated from human plasma as described previously (13). Other CSPs, such as ih-C4b, ih-C3b, ih-iC3b, and ih-C3d and complement proteins, like human C1 complex, native C4, C4BP, and factor I were purchased from ComplementTech (Tyler, TX, USA).

### Biotinylation of CSPs

Purification from human plasma and thioester carbonyl-specific-biotinylation via amine-PEG2-biotin reagent of ih-C4d was carried out as previously described (13). Iodoacetyl-LC-biotin (Pierce) was used to biotinylate rh-C4d via its thioester cysteine as described in detail previously (13). Biotin-X-NHS (Calbiochem, San Diego, CA, USA) was used according to the manufacturer's protocol to biotinylate rh-C4Ad, rh-C4Bd, rh-C4d-OL4, rh-C3d, ih-C3d, ih-C4b, ih-C3b, and ih-iC3b via their primary amino groups.

### Retroviral Expression Cloning

BW cells were retrovirally transduced to express a cDNA library generated from moDCs (21). The resulting cell pool was incubated with a recombinant biotinylated C4Ad/C4Bd isotype chimera, termed C4d-OL4, exhibiting a L1101P substitution in a C4B isotypic background (OL4-sequence: L1101PSPVIH), followed by incubation with SA-PE as secondary reagent. Three rounds of flow cytometric sorting were performed using a FACSAria^TM^ cell sorter (BD Biosciences, San Jose, CA). Single cell clones were established from the resulting cell pool by limiting dilution culturing. Genomic DNA was isolated from a C4d-reactive single cell clone and PCR-amplification of the integrated DNA was performed as previously described (21). Retroviral cDNA inserts were cloned into the retroviral expression plasmid pCJK2 (22). The resultant plasmids were subjected to DNA sequencing (MWG Biotech AG, Ebersberg, Germany) and expressed in BW cells.

### Determination of K_D_ Value and Competition Assays

Saturation and competition assays were carried out as previously described (13). In brief, NRP1 expressing BW cells were incubated with increasing concentrations of monomeric rh-C4d followed by adding the monoclonal C4d antibody, and subsequently PE-conjugated goat anti-mouse IgG, for detection of C4d-binding by flow cytometry. The K_D_ was determined by using the Langmuir-binding isotherm equation for single-class 1:1 binding equilibrium based on data from respective saturation experiments. Because of the washing steps after each incubation stage of the cells, which, dependent on the rate of dissociation have the potential to perturb the binding equilibrium, the K_D_ determined is an apparent affinity, not a true intrinsic affinity. Competition experiments were done by co-incubating biotinylated rh-C4d with unlabeled C4d or a polyclonal NRP1 antibody. Detection of receptor-bound biotinylated C4d was performed using SA-PE.

### ELISA

ELISA assays were carried out as previously described (13). In brief, ELISA plates were coated with 465 nM unlabeled CSPs overnight at 4°C. After washing and blocking, wells were incubated with rh-Ctrl-Ig, rh-NRP1-Ig (c.), rh-CRIg-Ig, rh-NRP1-CUB-Ig, or rh-NRP1-Ig fusion proteins (10 μg/ml each). Detection of bound Fc-fusion proteins was performed using an AP-labeled anti-human IgG-Fcγ-specific antibody and p-nitrophenyl phosphate disodium hexahydrate (Sigma-Aldrich) diluted in diethanolamine or with an HRP-conjugated goat anti-human IgG Fcγ-specific antibody and ABTS solution (Roche Applied Sciences, Penzberg, Germany). For solid-phase-based assays mimicking the natural deposition of C4d, plates were coated with or without streptavidin (+SA; –SA) at 100 μg/ml. After blocking, thioester cysteine-biotinylated ih-C4d (ih-C4d-bio; 465 nM) was incubated followed by washing steps. Wells were then incubated with rh-NRP1-Ig, rh-ILT4-Fc, rh-CTLA-4-Ig (3 μg/ml each), or monoclonal C4d antibody (at 2 μg/ml). Detection of bound fusion proteins was obtained using an AP-labeled anti-human IgG (Fcγ-specific) antibody and immobilized C4d with an HRP-labeled anti-mouse IgG (Fcγ-specific) antibody. The AP signal was assessed as described above. Optical density (OD) was assessed at λ = 405 nm using a microplate reader (Thermomax; Molecular Devices, Sunnydale, USA, CA).

### Natural Complement Deposition on Sheep Erythrocytes and Jurkat Cells

Natural complement deposition of C4d was performed as previously described (13). In brief, amboceptor-sensitized sheep erythrocytes (EA) were incubated with complement proteins obtained from ComplementTech in sequential order of C1-complex, C4 and C4BP and factor I. All steps were performed in gelatin veronal buffer (GVB) supplemented either with calcium chloride and magnesium chloride or EDTA. C4d deposition was confirmed using a C4d-specific monoclonal antibody. Subsequently, EA were incubated with rh-ILT4-Ig and rh-NRP1-Ig followed by appropriate secondary reagents and analyzed via flow cytometry. Natural complement activation on Jurkat ctrl and Jurkat CD40L cells was carried out by incubating 1 × 10^6^ cells with 5 μg/ml THG (rabbit anti-thymocyte globulin; Genzyme) and human serum (in final dilution of 1/20) for 2 h at 37°C.

### Generation of Cell Lines

BW cells were retrovirally transduced to express either the wildtype NRP1 or one of the following NRP1 deletion variants (a2b1b2, a1b1b2, a1a2b2, a1a2b1, a1a2, b1b2, and b1), all of which express the MAM/dimerization domain, the transmembrane segment and the cytoplasmic tail. In addition, BW cells expressing murine NRP1 (mNRP1) and ILT4 were generated. For activation of moDCs, Jurkat cells were retrovirally transduced to express CD40L. Wildtype NRP1, NRP1 deletion variants, mNRP1, ILT4, and CD40L were cloned into the retroviral expression vector pCJK2 (22) and cells were retrovirally transduced to express these molecules as described (21). BW cells and Jurkat cells were flow-sorted for surface expression of the respective molecules.

### Differentiation and Maturation of Monocyte-Derived Dendritic Cells (moDCs) in the Presence of CSPs

Peripheral blood mononuclear cells (PBMCs) were isolated from heparinized whole blood of healthy volunteer donors purchased from the Austrian Red Cross by standard density-gradient centrifugation with Lymphoprep (Axis-Shield PoC AS, Oslo, Norway). Donors gave their informed consent and approval was obtained from the ethics committee of the Medical University of Vienna (ECS1183/2016). Monocytes were isolated and differentiated into monocyte-derived dendritic cells (moDCs) as previously described (13, 23). MoDCs were activated by adding Jurkat cells expressing of CD40L for 24 h in the presence or absence of natural complement deposition. CSPs were deposited by incubating Jurkat control cells and Jurkat cells expressing CD40L with thymoglobulin (5 μg/ml) and human serum (1/20 dilution in full medium). NRP1 on immature DCs was blocked with a polyclonal NRP1 antibody or the respective isotype control.

## Results

### Neuropilin-1 (NRP1) Interacts With C4d

**Figure 1:**
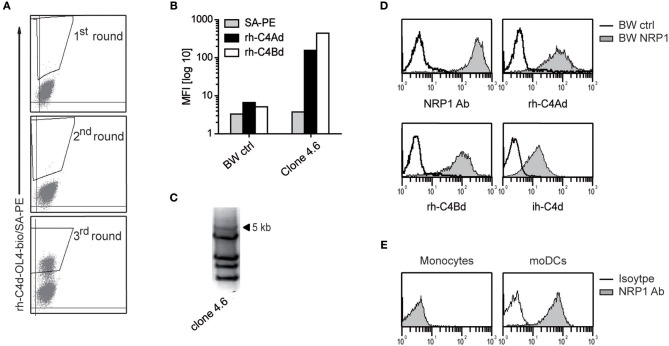
Identification of NRP1 as a receptor for C4d. **(A)** C4d-reactive cells enriched from a BW cell pool expressing a moDCs-cDNA library by multiple rounds of cell sorting. Sorting gates are shown. **(B)** A single cell clone derived from the C4d-reactive BW cell pool was probed with rh-C4Ad and rh-C4Bd and analyzed via flow cytometry. **(C)** PCR-amplification of retroviral inserts of a C4d-binding clone. **(D)** BW cells expressing a 5 kb retroviral insert encoding NRP1 were probed with a NRP1 mAb (monoclonal) or biotinylated rh-C4Ad, rh-C4Bd or ih-C4d (20 μg/ml each; open histograms: reactivity of NRP1 mAb or C4d to BW control cells; gray histograms: reactivity of NRP1 mAb or C4d to BW NRP1 cells). Biotinylation of rh-C4Ad and rh-C4Bd employed the NHS-biotin procedure, except for ih-C4d, which was specifically biotinylated on the thioester carbonyl moiety employing amine-PEG2-biotin reagent. **(E)** Monocytes and moDCs analyzed for NRP1 expression (open histograms: isotype control; gray histograms: NRP1 mAb). MFI, mean fluorescence intensity.

Using recombinant C4Bd, we previously identified ILT4 and ILT5v2 as receptors for C4d by screening an expression library derived from human monocyte-derived dendritic cells (moDCs) (13). Additional screening experiments using a C4Ad/C4Bd hybrid variant, termed C4d-OL4, as bait, yielded C4d-reactive cells (Figure 1A). Single cell clones were established from the C4d-reactive cell pool by limiting dilution and retroviral cDNA inserts were amplified from genomic DNA derived from a clone that was strongly reacting with C4d (Figures 1B,C). Re-expression of a cDNA of 5 kb in BW cells conferred reactivity to rh-C4Ad and rh-C4Bd as well as C4d isolated from human plasma (ih-C4d) (Figure 1D). DNA sequencing revealed that the cDNA encoded neuropilin-1 (NRP1, also known as CD304 and BDCA4) and expression of NRP1 was confirmed by flow cytometry (Figure 1D). NRP1 is a highly conserved transmembrane protein, which is involved in a variety of processes ranging from neuronal development, angiogenesis, and tumorigenesis to immunity (15, 16, 24–26). We demonstrated earlier that monocytes and moDCs both bound C4d (13). Therefore, we assessed NRP1 expression on these cells and found NRP1 to be absent on monocytes, whereas it is homogenously expressed on moDCs (Figure 1E). Moreover, we observed that NRP1 is being rapidly upregulated during monocytes differentiation toward moDCs *ex vivo* (Figure S1).

### Binding of Soluble CSPs to NRP1

**Figure 2:**
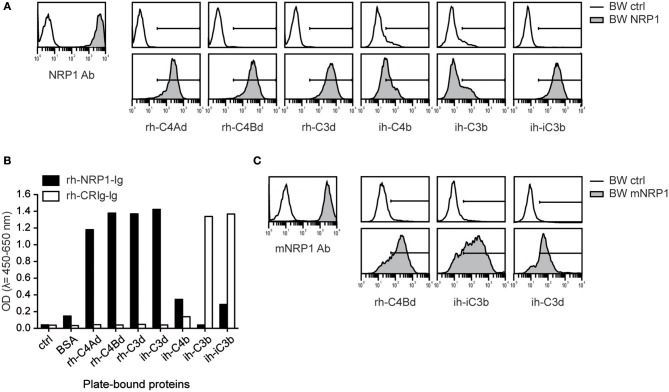
Interaction of NRP1 and mNRP1 with complement split products C4d, C3d, and iC3b. **(A)** Flow cytometric analysis of BW cells transduced to express high levels of human NRP1. Interaction of indicated CSPs (20 μg/ml each) with BW control cells (open histograms) and BW cells expressing NRP1 (gray histograms). Expression of NRP1 was verified with a monoclonal NRP1 antibody. **(B)** Interaction of plate-bound CSPs (465 nM each) with soluble recombinant human NRP1-immunoglobulin fusion protein (rh-NRP1-Ig) and complement receptor Ig fusion protein (rh-CRIg-Ig) analyzed in an ELISA-based assay. **(C)** Binding of murine NRP1 (mNRP1) mAb and recombinant human CSPs (rh-C4d, ih-iC3b, and ih-C3d) to BW control cells (open histograms) and BW cells expressing mNRP1 (gray histograms). Data shown is representative of two independently performed experiments.

Since complement receptors commonly bind several ligands, we assessed whether NRP1 would also bind to additional C3- and C4-derived CSPs. We generated BW cells expressing high levels of NRP1 and probed them with recombinant human or isolated human C4Ad, C4Bd, C3d, C4b, C3b, and iC3b. These experiments showed that NRP1, in addition to human C4d of both isotypes, strongly bound rh-C3d and ih-iC3b, whereas only weak binding was detected for ih-C4b and ih-C3b (Figure 2A). These interactions of soluble CSPs with NRP1 expressed on a cellular surface could be confirmed in a solid-phase assay applying rh-NRP1 immunoglobulin fusion protein (rh-NRP1-Ig) to immobilized CSPs (Figure 2B). Recombinant human complement receptor of the Ig superfamily (rh-CRIg-Ig) was found to interact with its established ligands, while no interaction with C4d was observed (Figure 2B). To test a potential interaction of CSPs with murine NRP1 (mNRP1), we generated BW cells expressing high levels of mNRP1 and analyzed the binding of rh-C4d, ih-iC3b, and ih-C3d. The results of these experiments confirmed that mNRP1 also acts as a receptor for CSPs (Figure 2C).

### Classical Receptor-Ligand Interaction Between NRP1 and C4d

**Figure 3:**
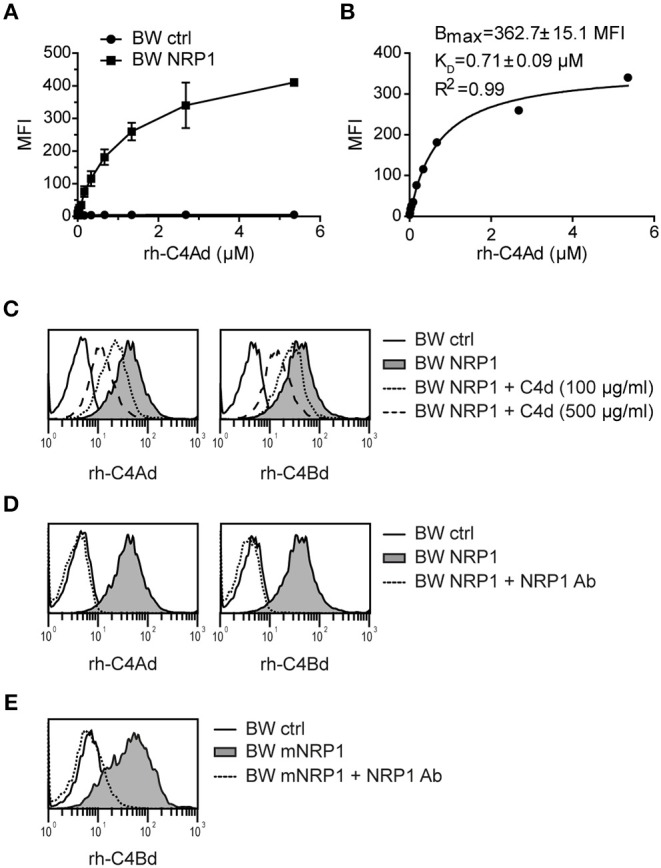
Classical receptor-ligand interaction between C4d and NRP1. **(A)** BW NRP1 cells or BW control cells were incubated with increasing concentrations of rh-C4Ad. Cell-bound rh-C4Ad was measured by flow cytometry using a monoclonal C4d antibody for detection. Mean and standard deviation of triplicate measurements are shown. **(B)** Dissociation constant (K_D_) of the C4d-NRP1 interaction was assessed via Langmuir binding isotherm equation for single class 1:1 binding equilibrium based on data shown in **A**. Error estimates of the fitted parameters to the mean of triplicate data points, and the correlation coefficient of the fit to the model are indicated. **(C,D)** Binding of biotinylated rh-C4Ad or rh-C4Bd (20 μg/ml each) to BW control cells or BW NRP1 in absence or presence of unlabeled rh-C4d **(C)** or a polyclonal NRP1 antibody (10 μg/ml) **(D)**. **(E)** Binding of biotinylated rh-C4Bd (20 μg/ml each) to BW control cells or BW mNRP1 in absence or presence of a polyclonal NRP1 antibody (10 μg/ml). In **(A–E)** probe biotinylation was performed with iodoacetyl-LC-biotin specifically targeting the thioester cysteine. MFI, mean fluorescence intensity.

In order to investigate the binding affinity between C4d and NRP1, BW cells expressing NRP1 and BW control cells were incubated with increasing concentrations of C4Ad (Figure 3A). Binding of C4Ad to BW NRP1 cells was dose-dependent and saturable and an apparent K_D_ value of 0.71 μM (±0.09 μM) was calculated from the binding curve (Figure 3B). Because of the potential of the washing steps in a flow cytometry-based binding assay to perturb the equilibrium toward dissociation, the apparent K_D_ determined from the measurements represents a minimal estimate of the intrinsic binding affinity.

In competition assays used to demonstrate specificity of the binding interaction, we could show that binding of biotinylated-C4d to NRP1 expressing cells was inhibited by the addition of an excess of unlabeled C4d (Figure 3C) or by a polyclonal NRP1 antibody (Figure 3D). C4d binding to murine NRP1 expressing cells was also blocked by adding a polyclonal NRP1 antibody that shows cross-reactivity toward murine NRP1 (Figure 3E). Taken together, these data indicate that the interaction of C4d with NRP1 bears the characteristics of a classical receptor-ligand interaction.

### NRP1 Interacts With Naturally Deposited C4d

**Figure 4:**
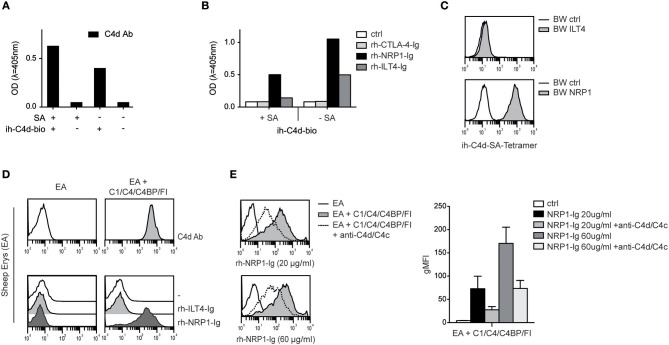
Interaction between NRP1 and C4d deposited to surfaces via thioester binding following classical complement activation. **(A)** C4d biotinylated at its intrinsic thioester carbonyl via amine-PEG2-biotin reagent (ih-C4d-bio) was immobilized to a solid phase either via streptavidin (+SA) or in random orientation without SA (–SA) and detected by ELISA using a monoclonal C4d antibody. **(B)** Interaction between C4d, immobilized to a solid phase as shown in **(A)**, and rh-NRP1-Ig and rh-ILT4-Ig. Rh-CTLA-4-Ig fusion proteins and goat anti-human-IgG (ctrl) served as negative controls. **(C)** C4d biotinylated via its intrinsic thioester was tetramerized using streptavidin-APC and probed with BW cells expressing ILT4 or NRP1 and analyzed via flow cytometry. **(D)** Antibody-coated sheep erythrocytes (EA) bearing C4d were prepared using purified C1, C4, C4 binding protein (C4BP), and factor I (FI) as described in Material and Methods. Deposition of C4d was detected with a monoclonal C4d antibody (upper panel). Interaction between C4d-loaded EA and control EA with immunoglobulin fusion proteins (rh-ILT4-Ig; rh-NRP1-Ig) is shown in the lower panel. **(E)** Binding of goat anti-human-IgG (ctrl) or rh-NRP1-Ig (20 or 60 μg/ml) to C4d-EA in the presence or absence of an anti-C4d and anti-C4c antibodies. Data shown are representative for two independently performed experiments. gMFI, geometric mean of fluorescence intensity.

Our group recently identified ILT4 as a cellular receptor for soluble complement fragment C4d, however binding of ILT4 to naturally deposited C4d via classical complement pathway activation could not be observed (13). Thus, the ILT4 binding site of C4d does not seem to be available on physiologically oriented C4d after thioester-mediated covalent binding to a given surface. Therefore, ILT4 may not function as a “classical” receptor for C4d and other CSPs immobilized on surfaces during complement activation (13). Consequently, we set out to assess the capacity of NRP1 to bind to C4d and other CSPs deposited in their natural orientation. First, we performed experiments where we biotinylated C4d specifically via the carbonyl group of its intrinsic thioester and determined the binding of rh-NRP1-Ig and rh-ILT4-Ig to this protein immobilized either in a random fashion or via streptavidin, resulting in an orientation mimicking C4d generated on surfaces during complement activation (see M&M for details). Using a monoclonal C4d-specific antibody, we confirmed the presence of immobilized C4d in both conditions (Figure 4A). Whereas, rh-ILT4-Ig did not bind to C4d immobilized in its natural orientation, we could readily detect rh-NRP1-Ig binding, indicating that NRP1 interacts with naturally deposited C4d (Figure 4B). In line with these findings, we observed that C4d tetramers, generated by using C4d biotinylated via its thioester and streptavidin-APC, strongly bound to BW cells expressing NRP1, whereas a specific interaction with BW ILT4 was not observed (Figure 4C). Moreover, in experiments in which large amounts of C4d were deposited on sheep erythrocytes via classical complement activation (see M&M for details), strong binding of rh-NRP1-Ig, but not rh-ILT4-Ig to the C4d-loaded erythrocytes was detected via flow cytometry (Figure 4D). Rh-NRP1-Ig binding to complement activated sheep erythrocytes was specifically blocked by anti-C4d and anti-C4c antibodies (Figure 4E). Taken together these experiments indicate that NRP1 functions as a receptor for CSPs physiologically deposited on surfaces during complement activation.

### CSPs Interact With the b1-Region of NRP1

**Figure 5:**
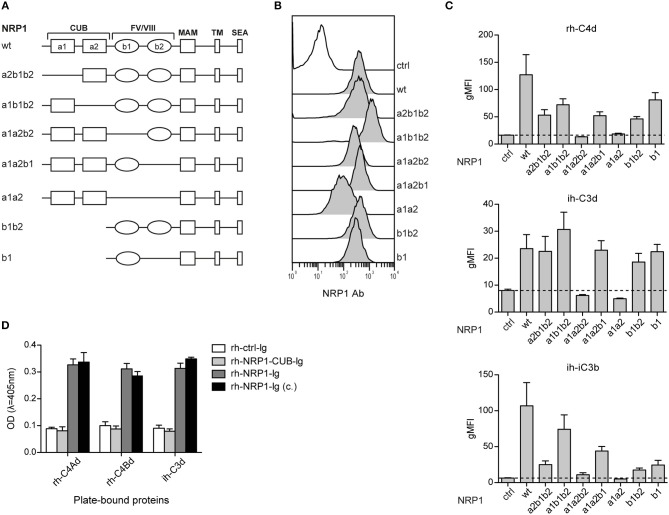
The b1 region of neuropilin-1 is essential for the interaction with CSPs. **(A)** Schematic representation of wildtype (wt) NRP1 and seven deletion variants. **(B)** Flow cytometric analysis of BW cells expressing wildtype NRP1 and NRP1 deletion variants depicted in **(A)** using a polyclonal NRP1 antibody. **(C)** BW control cells (ctrl) and BW cells expressing wt-NRP1 or NRP1 deletion variants were probed with the indicated biotinylated rh-C4Bd, ih-C3d, and ih-iC3b molecules (20 μg/ml each) followed by SA-PE and analyzed via flow cytometry. The data are from three independent experiments performed in triplicates. **(D)** Binding of an immunoglobulin fusion protein representing the CUB domain (a1a2) of NRP1 (rh-NRP1-CUB-Ig; 10 μg/ml) and two differently generated fusion proteins representing the extracellular part (a1a2 and b1b2) of NRP1 (rh-NRP1-Ig (generated in house) and rh-NRP1-Ig (c.) (commercial); 10 μg/ml each) to immobilized rh-C4Ad, rh-C4Bd and ih-C3d (465 nM each) was assessed by ELISA. gMFI, geometric mean of fluorescence intensity; SEA, the SerGluAla C-terminal tripeptide represents a PDZ domain-binding motif.

Neuropilin-1 is a type 1 transmembrane glycoprotein of 923 amino acids with a large extracellular region and a short cytoplasmic tail, the latter containing a PDZ binding motif that may localize NRP1 to signaling components in the membrane. Several distinct domains are contained in its extracellular region: two CUB (complement binding factors C1s/C1r, Uegf, BMP1) domains (a1/a2), two coagulation Factor V/VIII homology domains (b1/b2) and a MAM (homologous to meprin protease, A5 antigen, receptor tyrosine phosphatase μ and K) (c) domain (Figure 5A). In order to identify the structural features required for NRP1-CSPs interaction, we designed seven NRP1 constructs harboring distinct deletions in the CUB and FV/VIII homology domain (Figure 5A). These constructs were retrovirally expressed in BW cells and surface expression was confirmed for all constructs via flow cytometry using a polyclonal NRP1 antibody (Figure 5B). While the binding of ih-iC3b, ih-C3d, and rh-C4Bd to NRP1 derivative molecules lacking the b1 region was completely abolished, at least in the case of ih-C3d and rh-C4Bd, deletions of a1, a2, and/or b2 had little effect on the binding (Figure 5C). By contrast, for both ih-C3d and rh-C4Bd surface expression of the b1 domain on its own was sufficient for strong binding of these ligands. For the larger ligand ih-iC3b, in addition to dependence on the presence of the b1 Factor V/VIII homology domain, there is some indication that the a1 CUB domain may also contribute (Figure 5C, bottom panel), but it needs to be pointed out that the a1b1b2 construct that most clearly suggests this is also expressed at the highest level of any of the constructs (Figure 5B). Furthermore, by applying an ELISA approach, we could confirm for rh-C4Ad, rh-C4Bd, and ih-C3d that the CUB domains on their own did not mediate measurable binding as an immunoglobulin fusion protein representing a1/a2 of human NRP1 (rh-NRP1-CUB-Ig), but lacking the b1 and b2 coagulation factor V/III homology domains, failed to interact with immobilized CSPs. By contrast, two independently generated NRP1-Ig constructs [rh-NRP1-Ig; rh-NRP1-Ig (c.) (see Materials and Methods section for details)] harboring the CUB and Factor V/VIII homology domains, but lacking the meprin domain, were readily bound to rh-C4Ad, rh-C4Bd, and ih-C3d (Figure 5D). Taken together, our data strongly suggest that the dominant interaction between the TED domains of the CSPs and NRP-1 is with the b1 coagulation FV/FVIII homology domain.

### NRP1 Binds to MHC Class I Molecules

**Figure 6:**
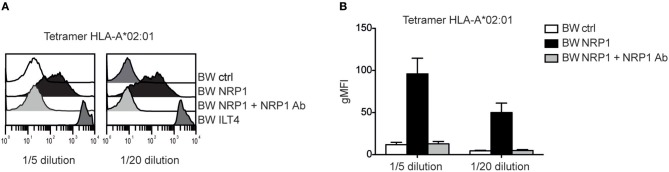
NRP1 interacts with MHC class I molecules**. (A)** Binding of tetramers representing MHC class I molecule HLA-A*02:01 (CMV CLGGLLTMV) with BW control cells and BW cells expressing NRP1 or ILT4 in the presence or absence of a polyclonal NRP1 antibody via flow cytometry. **(B)** Data of two independently performed binding experiments is shown. gMFI, geometric mean of fluorescence intensity.

Although NRP1 and ILT4 belong to different molecular families and do not share structural similarities, they both interact with C4d and other CSPs. Since ILT4 is an established receptor for MHC class I molecules (27), we assessed a potential interaction of NRP1 with these molecules and probed BW cells expressing high levels of ILT4 or NRP1 with different MHC class I tetramers. MHC class I tetramers bound to NRP1 expressing cells but with a clearly more heterogeneous profile and a seemingly weaker extent than to cells expressing ILT4 (Figure 6 and Figure S2). This binding was fully blocked by the addition of a polyclonal NRP1 antibody.

## Discussion

NRP1 is a highly conserved type 1 transmembrane protein that was originally described as a neuronal adhesion molecule (28–30). The receptor is defined by its large extracellular part with several distinct domains that can act both independently and cooperatively to mediate various functions in neuronal development and axonal guidance. NRP1 has a short cytoplasmic tail and functions by forming a complex with class A plexins after binding to its ligand, the class 3 semaphorins, specifically Sema3A (31). NRP1 is also a high-affinity receptor for VEGF, and this interaction was shown to exert important functions in angiogenesis and cardiac development (32). On endothelial cells, NRP1 forms a complex with the VEGFR2, which mediates downstream signaling upon VEGF binding (33, 34). In addition to semaphorins and VEGF, NRP1 was described as a receptor for several other ligands including platelet derived growth factor (PDGF) (35).

NRP1 is expressed on many cells of the immune system, including DCs, macrophages, mast cells, and basophils (36–38). Additionally, it serves as a marker for plasmacytoid DCs and murine, but not human, regulatory T cells (mTregs). Studies in murine models indicated that NRP1 is involved in tolerance induction and contributes to the suppressive function and stability of Tregs (39, 40). Discordant expression of NRP1 on murine vs. human immune cells, especially in Tregs (41), and the lack of robust *in vitro* assays evaluating the function of this receptor, makes it challenging to understand the functional role of NRP1 in human immunity. In the nervous and vascular system, NRP1 mediates its functions in a co-receptor and ligand-dependent manner. Currently, it is not known whether in the immune system, NRP1 functions independently or also as part of a receptor complex. NRP1-NRP1 interactions between T cells and DC in the immunological synapse have been proposed to mediate the initiation of primary human T cell responses (36). Recently, semaphorin 4a (Sema4A) was described as a novel NRP1 ligand critically involved in suppression of anti-tumor responses by mTregs, but additional ligands mediating immune-modulatory effects of NRP1 are not well-defined (39).

Using an unbiased screening approach, we identified C4d and other CSPs as novel ligands for NRP1. In an earlier study, we identified ILT4 as the first receptor for complement molecule C4d, using a similar strategy. ILT4 was shown to additionally bind C3d, C3b, and iC3b, which are ligands for classical complement receptors, but unlike these receptors, ILT4 only bound soluble CSPs and did not appear to interact with complement proteins naturally deposited to surfaces during complement activation (13). In the present study, we show a series of experiments that indicate that in contrast to ILT4, NRP1 binds to naturally deposited C4d, and other CSPs, thus behaving as a classical receptor for CSPs. We further found that the b1 domain of the coagulation Factor V/VIII homology domains, which mediates interaction between NRP1 and VEGF (42, 43), is crucial for the binding of NRP1 with complement proteins.

The respective CSP fragment binding profiles of ILT4 and NRP1 indicate that in both cases C3d and C4d constitute the minimal binding fragment. However, the location of the binding sites for ILT4 and NRP1 on the respective surfaces of the TED domains comprising C3d and C4d must clearly be different. TED domains are dome-shaped structural entities having on one side a convex surface containing the thioester-forming residues mediating covalent binding, and a concave surface on the opposite side (20, 44). The binding of ILT4 to soluble CSPs, but not to CSPs either covalently-deposited on targets in the course of complement activation, or physiologically oriented on plastic via thioester-specific biotinylation and capture (13), strongly suggests that the ILT4 binding site is located on the convex surfaces of the TED domains, and in the vicinity of the thioester bond-forming residues, as this surface would be sterically occluded as a result of binding to the target. The fact that NRP1 is able to interact with CSPs bound to targets in the physiologically relevant orientation clearly excludes this area of the respective TED domains as mediating the binding interaction. However, two plausible, and non-mutually exclusive locations of the NRP1 binding site(s) elsewhere on the TED domains are hinted at by the binding preference of NRP1 for C4d, C3d, and iC3b, but not for the primary split products C4b and C3b. For the first possibility, at least with respect to the C3 fragments, this is the same fragment binding hierarchy previously observed for the interaction with CR2 (45). An identical fragment binding hierarchy has also been observed for the interactions with the C3-binding fragments of the *Staphylococcus aureus* evasion molecules referred to as Sbi-IV and Efb-C (46, 47). X-ray co-crystal structures of C3d with each of these three ligands, although differing somewhat in the fine details of their interacting partner residues on C3d, nevertheless reveal a considerable overlap in their respective binding sites on the concave surface of the C3 TED domain (47–49). In particular, an important subregion of their respective binding sites is centered around an acidic cluster on the concave surface of the TED domain (D1029, E1030, E1032, prepro-human C3 numbering). In the X-ray crystal structure of C3b, this region of the TED domain makes contact with the MG1 domain of the C3 β-chain “keyring,” and would thus block an important part of the binding site for CR2, Sbi-IV, and Efb-C (50). In iC3b, the TED domain has been shown to move considerably away from the MG1 domain (51). We therefore suggest that the area of the concave face of the TED domain that makes the close approach to MG1 in context of the C3b fragment contributes at least a portion of the NRP1 binding interface on the C3 TED domain, as this site becomes fully accessible upon further proteolytic processing of C3b to first iC3b, and subsequently to C3d. Since the structure of C4b shows a very similar contact of the TED domain with the MG1 domain of the C4 β-chain “keyring” (6), by extension we propose that the corresponding region of the C4 TED domain contributes at least a portion of the binding interface for NRP1. As there is no stable iC3b equivalent CSP of C4, the site only become accessible following the factor I-mediated proteolytic cleavages that leaves C4d on the target surface.

The second plausible explanation for the fragment binding hierarchy derives from the fact that factor I cleavage of C3b and C4b would produce a C-terminal arginine in the TED domains of the CSPs iC3b, C3d, and C4d. This, in turn, relates to the known mode of binding of another NRP1 ligand, namely VEGFA, which, as for the CSPs, has also been localized to the NRP1-b1 domain. Structural and biochemical data have shown that the interaction between VEGFA and the so-called b1-binding pocket of NRP1 is dependent upon extensive contacts with the carboxy-terminal arginine residue (R164) (43). These contacts involve both a salt bridge of the guanido group to the D320 side chain of NRP1, as well as the terminal carboxylate group being involved in an extensive hydrogen bonding network with the side chains of NRP-b1 residues Y353, S346, and T349. Although these C-terminal arginine contacts are crucial to the binding, there are nevertheless additional contacts well-outside of the C-terminal residues which contribute to the overall binding affinity between NRP1-b1 and VEGFA, and indeed dictate the specificity of VEGFA for NRP1 over its homolog NRP2 (43). We would expect the same to be the case for iC3b, C3d, and C4d if a binding modality involving the factor I-generated C-terminal arginine of the respective TED domains was to be employed. Further experimental work will be required to determine whether either or both of the above hypothesized binding modalities for the interactions of iC3b, C3d, and C4d with NRP1-b1 are actually employed.

Human NRP1 has been implicated to play an important role in T-cell-DC interactions (36), but preliminary data suggest that CSPs-deposition on the Jurkat T cell line does not modulate CD40L-induced activation of monocyte-derived DCs (Figure S3). Recently, the NRP1 ligand Sema4A was reported to also bind to ILT4 (52). Our results indicate that MHC class I molecules, which are the classical ligands for ILT4, also interact with NRP1 revealing that ILT4 and NRP1 share three distinct types of ligands.

Further research will be necessary to reveal a potential functional significance of the CSP-NRP1 interaction, which at present is hampered by the lack of both reliable *in vitro* assays and a defined signaling pathway for this receptor. However, since we found murine NRP1 to interact with CSPs, it might be possible to assess a potential immunomodulatory effect of naturally deposited CSPs and murine NRP1 *in vivo*.

## Data Availability

The datasets generated for this study are available on request to the corresponding author.

## Ethics Statement

Written informed consent was obtained from the individual(s) for the publication of any potentially identifiable images or data included in this article.

## Author Contributions

CB, JH, PS, and WP: conceptualization and design of experiments. CB, JH, WP, AD, and JL: acquisition of experiments. CB, JH, WP, AD, JL, and PS: analysis and interpretation of data. JH, PS, WP, GZ, MW, and DI: resources. CB, JH, and PS: wrote the manuscript. CB, AD, WP, JL, MW, DI, GZ, PS, and JH: manuscript review, read, and approved.

### Conflict of Interest Statement

The authors declare that the research was conducted in the absence of any commercial or financial relationships that could be construed as a potential conflict of interest.

## Abbreviations

C4BP: C4b-binding protein
CRIg: complement receptor of the immunoglobulin family
CSP: complement split product
CUB: complement binding factors C1s/C1r
ILT4: immunoglobulin-like transcript 4
TED: thiol ester-containing domain.
