# Supplementary material for: Neuropilin-1 Acts as a Receptor for Complement Split Products
Source: Front Immunol. 2019 Sep 13;10:2209. doi: 10.3389/fimmu.2019.02209 (PMC6753332; doi:10.3389/fimmu.2019.02209)
Supplement: Supplementary file 1 [file Data_Sheet_1.docx]

**Supplementary Material**


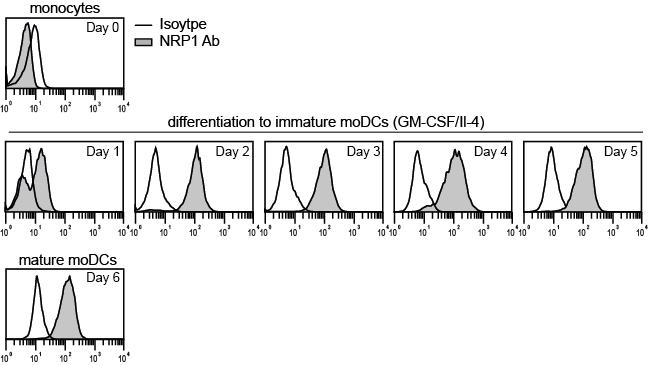


**Figure S1. Neuropilin-1 is upregulated during differentiation of monocytes to dendritic cells.**

A) Expression of NRP1 (grey histograms) on monocytes and cells differentiated towards immature monocyte-derived dendritic cells (immature moDCs) for five days by addition of GM-CSF and IL-4. Mature moDCs were generated by adding LPS (300 ng/ml) at day five to the immature moDCs for 24 h. Open histograms represent reactivity of isotype control antibody. Cell surface expression was assessed via flow cytometry.


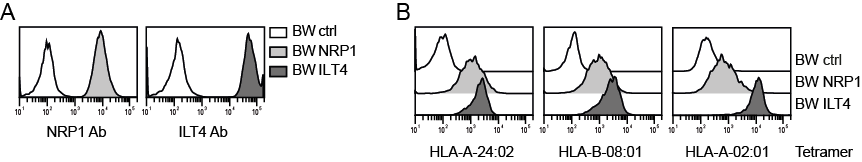


**Figure S2. NRP1 interacts with different MHC class I molecules.**

A) Flow cytometric analysis of BW cells transduced to express high levels of human NRP1 or ILT4. B) Binding of tetramers representing different MHC class I molecules HLA-A*24:02 (CMV QYDPYAALF), HLA-B*08:01 (CMV ELRRKMMYM) and HLA-A*02:01 (CMV LLFGVPVYV) to BW control cells and BW cells expressing NRP1 or ILT4. Ab, antibody


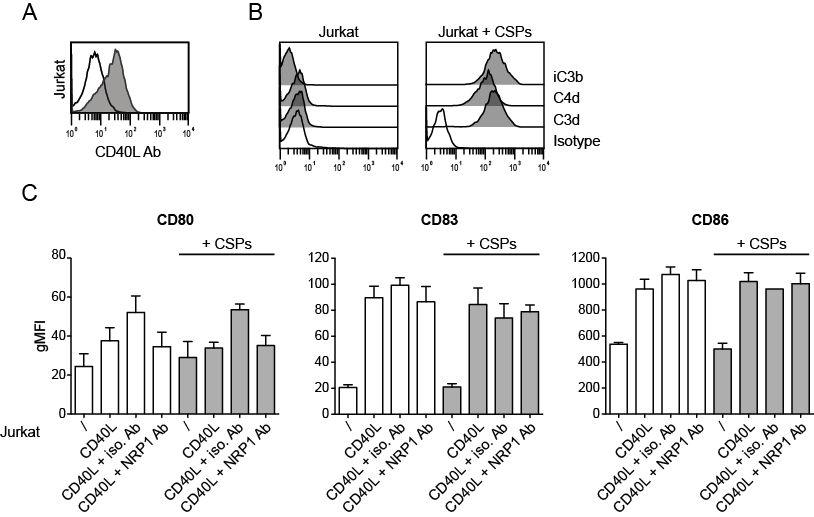


**Figure S3. CSPs-Neuropilin-1 interaction does not modulate the activation of DCs.**

A) Jurkat T cells (open histogram) and Jurkat T cells engineered to express CD40L (grey histogram) were probed with an CD40L antibody and analyzed via flow cytometry (B) Control Jurkat cells and Jurkat cells incubated with thymoglobulin and human serum to induce deposition of CSPs (Jurkat + CSPs) were probed with antibodies to C4d, C3d, and iC3b and analyzed via flow cytometry. (C) Day five immature moDCs were co-incubated with the indicated Jurkat cells in presence or absence of isotype control and polyclonal NRP1 antibody for 24h. Subsequently, moDCs were assessed for the expression of the activation markers CD80, CD83 and CD86. Results of two independently performed experiments with two donors are summarized. Ab, antibody; CSPs, complement split products
